# Podcast-driven insights into Charles Bonnet Syndrome: Impact on self-management strategies and communication

**DOI:** 10.1177/20552076251382830

**Published:** 2025-11-18

**Authors:** Bethany E Higgins, Deanna J Taylor, Sonali Dave, Sarah Sowerby, David P Crabb, Tamsin Callaghan

**Affiliations:** 1 Optometry and Visual Sciences, 4895 City St George's, University of London, London, UK; 2 WordBird, London, UK; 3 NIHR Royal Free Clinical Research Facility, Research and Development, 4965 Royal Free London NHS Foundation Trust, London, UK

**Keywords:** Charles Bonnet Syndrome, podcast, hallucination management, digital intervention, behaviour change

## Abstract

**Purpose:**

This study compared the effectiveness of two educational podcasts on behavioural responses to Charles Bonnet Syndrome (CBS) hallucinations. One podcast provided general information and management strategies, while the other included the same content but also encouraged participants to actively interact with their hallucinations (e.g. by touching them).

**Methods:**

In this double-masked, two-arm randomised comparative study, participants were recruited through charities and social media. All participants completed a baseline survey assessing hallucination frequency and severity. They were then randomised to receive either the education podcast or the interaction podcast. Approximately three weeks after listening, participants completed a follow-up survey. The primary outcome asked, ‘Have you made any changes to how you respond to your hallucinations since listening to the podcast?’ Secondary outcomes included which strategies participants had tried and their perceived helpfulness, whether they were inspired to try them, changes in hallucination characteristics, perceptions of the podcast's usefulness and enjoyability, and whether they had disclosed their symptoms to new people.

**Results:**

Fifty-four people with CBS (76% female; 70% aged >65 years) participated. Twenty-eight received the education podcast and 26 received the interaction podcast. Across the whole cohort, 80% reported no change in how they responded to hallucinations, with no statistically significant difference between the two podcast groups (*p* = 0.8). However, 52% felt inspired to try the strategies. Forty-eight percent found familiarising themselves with hallucinations helpful, while 33% found improving sleep ineffective. Sixty-four percent found the podcast useful, though only 42% found it enjoyable. Following the podcast, 63% told new people about their symptoms.

**Conclusions:**

No significant difference was found between the two podcast in altering behavioural responses. However, both encouraged uptake of self-management strategies, suggesting that podcasts may be a useful support tool for individuals with CBS. Further research is needed to evaluate long-term impact.

## Introduction

Charles Bonnet Syndrome (CBS) represents a phenomenon observed in individuals with visual impairment, characterised by the occurrence of visual hallucinations despite preserved cognitive function. While hallucinations have traditionally been described as complex, such as representing people or animals, contemporary definitions encompass a broader spectrum of visual manifestations, including simple patterns or flashes.^1^ Around 20% of individuals living with vision impairment are believed to experience CBS^2^ with visual impairment, advancing age and social isolation identified as a contributing risk factors.^3,4^ Despite its prevalence, CBS often remains underreported and poorly understood, including its management and psychosocial effect, necessitating further research in these areas.

The impact of CBS extends beyond presence of hallucinations, encompassing various aspects of affected individuals’ quality of life (QOL). While some individuals may perceive these hallucinatory experiences as benign, it is estimated a third of sufferers report a negative impact on their overall wellbeing.^5^ This negative outcome CBS, as characterised by Cox and ffytche, highlights the need for investigation to outline the determinants of QOL in people with CBS and to assess appropriate strategies people can use to help manage their condition. Medical treatments are being investigated,^6^ but can be complex, costly, and difficult to access. In contrast, the strategies explored in this study are simple, free, and easily self-applied.

The aim of CBS management is twofold: to reduce the frequency and severity of hallucinations where possible, and to minimise their negative impact on emotional wellbeing and QOL. Even when hallucinations persist, improved coping strategies may reduce distress and functional disruption, thereby enhancing overall QOL. Various self-management strategies have been proposed to mitigate hallucinations in CBS patients, including techniques such as closing the eyes or moving them from side to side, adjusting lighting conditions, and actively interacting with the hallucinations through touching or verbal communication. However, whilst these interventions are currently recommended by some,^7^ they have primarily stemmed from anecdotal evidence and case studies,^8^ lacking comprehensive assessment through rigorous scientific investigation. It remains unclear which of these interventions, if any, is most effective in managing CBS hallucinations, and some management techniques may be easier to implement than others. To ensure effective support for individuals with CBS, it is imperative that stakeholders prioritise evidence-based management approaches.

The provision of information on management and support for people with CBS is typically available in written format via websites or leaflets, despite the fact that patients with CBS have various forms of visual impairment. Podcasts are downloadable, electronic audio files that may be listened to on mobile phones, portable audio players (MP3 players) or computers.^9^ They represent a novel, cost-effective modality for disseminating health-based information, offering a versatile platform that is accessible and convenient, particularly for visually impaired consumers. Research indicates that educational podcasts have a positive influence on learning, self-efficacy, and attitudes across a range of subjects.^10^ Furthermore, podcasts have demonstrated potential as effective tools for health promotion, fostering high levels of engagement and influencing both knowledge and behaviours.^11^ Hence, podcasts have the potential to deliver information on management strategies to individuals with CBS, serving to educate them about their condition and empower them to engage in self-management practices.

The primary objective of this double-masked two-arm randomised comparative study was to evaluate the impact of two different podcast-based educational approaches on how individuals respond to their CBS hallucinations. Specifically, the study aimed to assess whether an interaction podcast, which encouraged active engagement with hallucinations (e.g. interacting physically with the hallucinations), would lead to different behavioural responses compared to an educational podcast that provided general information on CBS management strategies without explicitly promoting direct interaction. It was hypothesised that participants who received the interaction podcast would be more likely to adopt active engagement strategies compared to those who received the educational podcast. Additionally, the study explored whether either podcast influenced a change in the characteristics of hallucinations (e.g. frequency and duration), whether participants found the podcast useful and/or enjoyable, and if they had spoken to new people about their symptoms.

## Methods

### Study setting

This study took place at City St George's, University of London, and in the homes of participants. Recruitment, randomisation, and administration of study materials were coordinated through the university, while participants accessed the podcasts and questionnaires remotely from their own homes.

### Participant recruitment

Participants were recruited from May 2022 to April 2023 through social media adverts and through the help of national and local vision-related charities including the Macular Society, Esme's Umbrella, RNIB and Lincoln and Lindsey Blind Society (see Acknowledgements), as part of a broader study on the psychosocial impact of CBS.^12^ Participants had to be currently experiencing CBS (self-reported), aged ≥18 years, have impaired vision (regardless of cause), not report cognitive impairments or a history of Parkinson's disease, and have completed the baseline questionnaire in the initial study. No formal diagnosis of CBS by a clinician was necessary for inclusion and any form of visual hallucination (both simple and complex) were accepted.

### Patient and public involvement

Before initiating the study, patient and public involvement (PPI) activities were conducted to gain insights into the experiences of individuals with hallucinations. In the development of the podcast, authors sought expertise from individuals familiar with CBS and visual impairment. A psychiatrist and a patient with CBS were invited to be interviewed on the podcast, contributing their perspectives and ensuring that the podcast content resonated authentically with the experiences of those living with the condition. Additionally, to maximise accessibility, a focus group was held with two visually impaired people with age-related macular degeneration (AMD) who offered feedback on questionnaire design and accessibility.^12^ Furthermore, the charities Macular Society and Bravo Victor reviewed the online baseline and follow-up questionnaires to ensure compatibility with popular screen readers, including NonVisual Desktop Access (Assistivlabs, Delaware, USA) and Job Access with Speech (Freedom Scientific, Florida, USA). Feedback confirmed the survey was readable using these tools. For individuals without computer access or who preferred not to complete the questionnaires on paper, a researcher (BH) was available to administer it by telephone.

### Double-masked randomised comparative protocol

Following an eligibility assessment, participants were randomly assigned to either the interaction podcast or the educational podcast group by a member of the research team (SD) on a 1:1 allocation. To maintain the integrity of the masking process, the designated podcast type and format were sent separately from the team member responsible for data collection and analysis (BH). This ensured that neither the participants nor the analysts were aware of group allocations during the data collection phase. Upon completion of data collection, data were unmasked.

### Podcast development and delivery

The two podcasts were co-created by a multidisciplinary team comprising members of the research team (BH, DT and TC), a psychiatrist, a patient who has personal experience with visual impairment and CBS, and Wordbird, a UK-based healthcare communications agency. Wordbird recorded the podcasts pro bono, with an experienced narrator ensuring smooth flow and coherence on the recording day. The podcasts were recorded using high-quality microphones and recording software in two primary locations: the offices at WordBird and a hospital office. Editing of the recordings was performed by WordBird, and post-production checks were conducted by the research team and department colleagues at City St George's, University of London to ensure audio clarity and adherence to study objectives.

**Figure 1.**
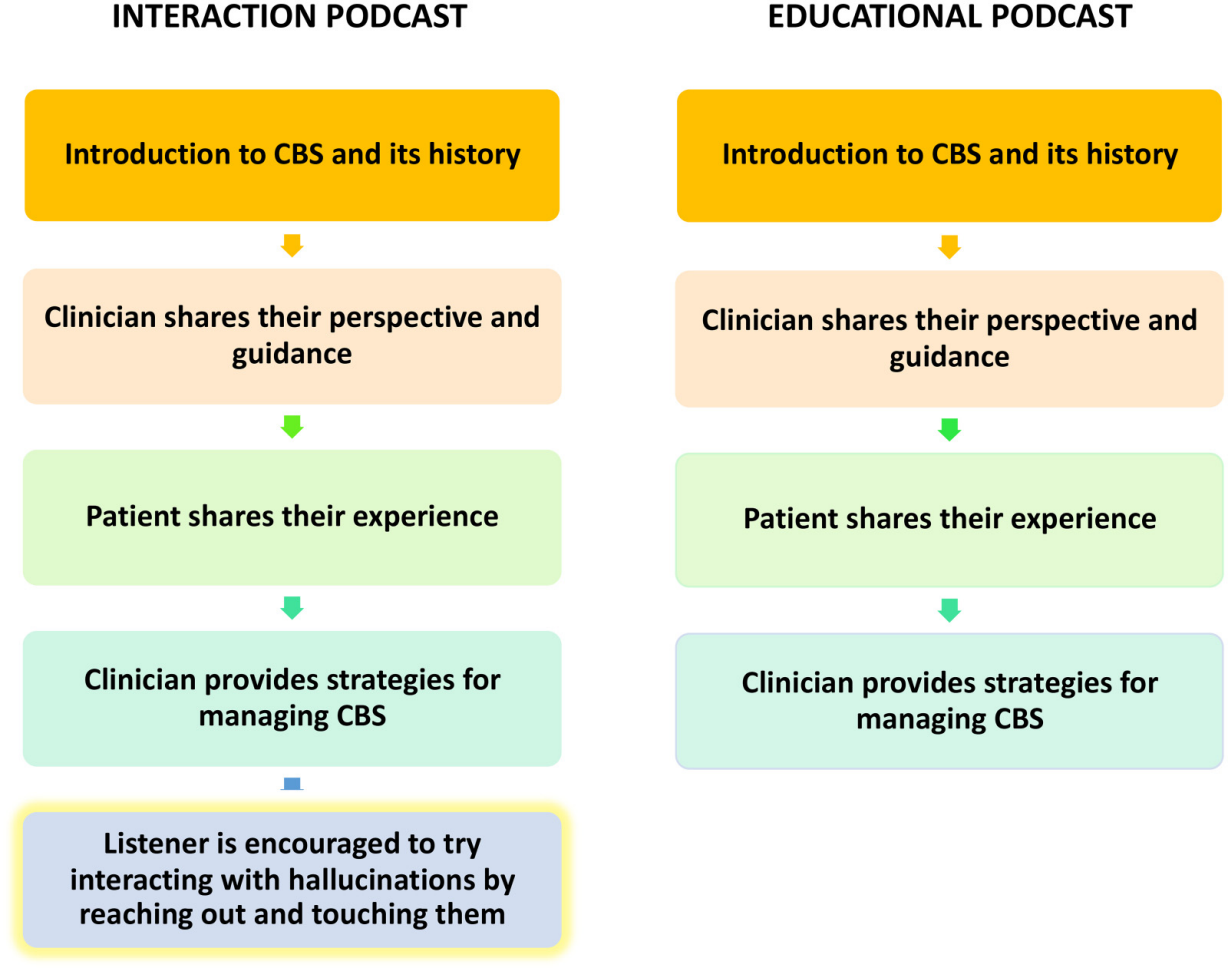
Two flowcharts illustrating the timeline of topics covered by both podcasts, and similarities and differences between the two.

*Note*: Both podcasts are identical, apart from the additional segment at the end of the interaction podcast, and were of a similar length.

Both versions of the podcast (interaction and educational) were ∼15 min long (15 min 51 s and 15 min 10 s, respectively) and consisted of near identical content. The podcasts began with an introduction to CBS and its historical background. The psychiatrist offered insights into CBS from a clinical perspective, while the patient shared their personal experiences with the phenomenon. Concluding the podcasts, the clinician provided potential strategies for self-managing CBS. The management techniques included: environment adjustments (e.g. adjusting lighting), mental engagement (e.g. distraction techniques), sleep improvement, familiarisation with hallucinations (e.g. naming them, recognising recurring features), reality testing (e.g. reminding oneself the hallucination isn’t real) and interaction techniques (e.g. attempting to disrupt them through touch). The interaction podcast featured an additional 41 s segment where the clinician highlighted the potential benefits of interacting with hallucinations, suggesting it as a strategy to mitigate episodes. The narrator then reinforced this notion, urging listeners to consider engaging with their hallucinations during future occurrences by trying to reach out and touch them. This segment was not included in the educational podcast (see Figure 1).

**Figure 2.**
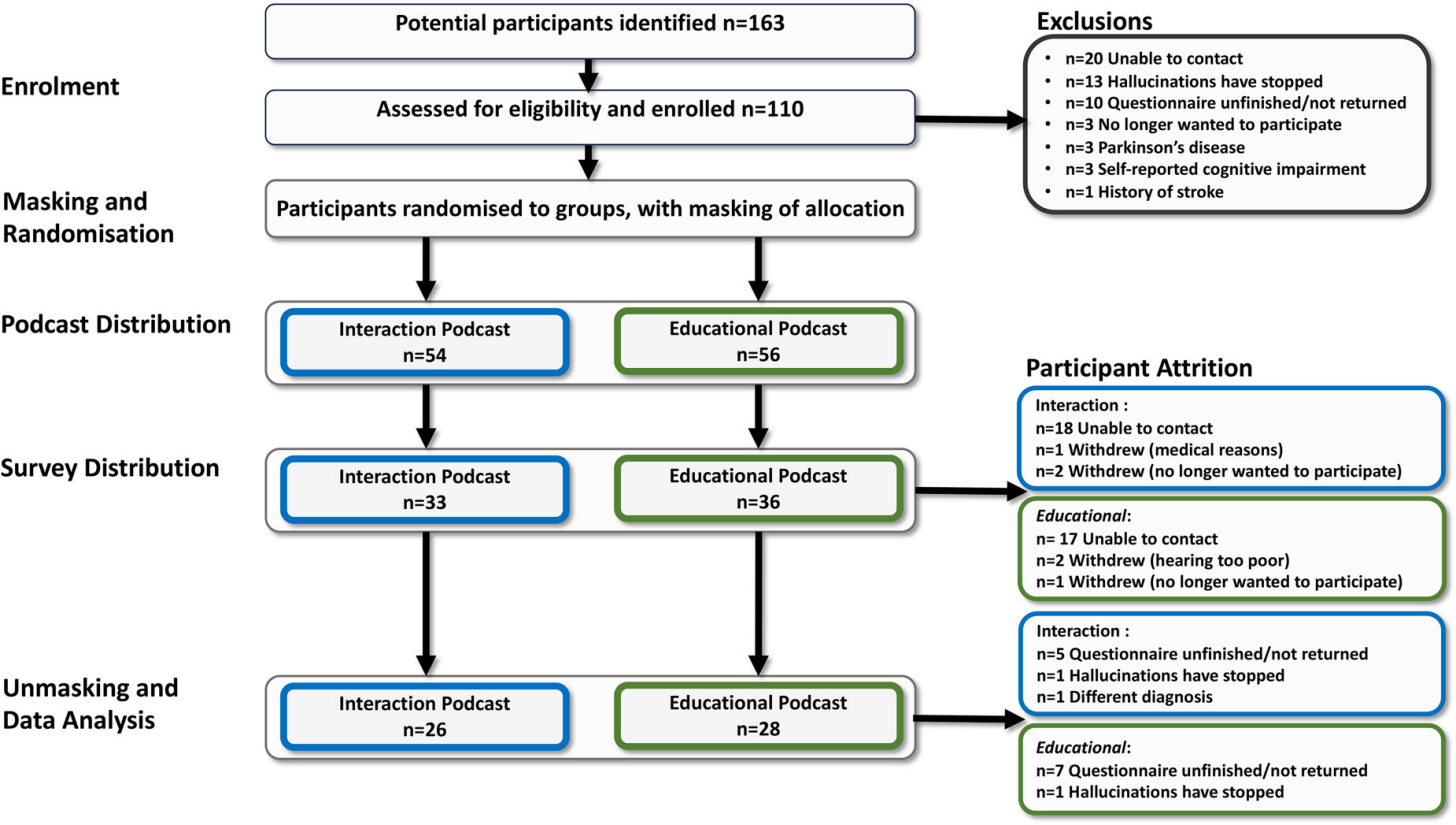
Flow diagram of protocol followed and participant attrition.

The podcasts were shared with the participants via online link, CD, USB stick or MP3 player, compatible with audiobook players, depending on participant preference. Participants were asked to listen to the podcast at least once but were encouraged to listen to it as many times as they wanted. They were given a three-week window with the podcast and were then sent the follow-up questionnaire. This timeline was deemed sufficient, as over 90% of recruited participants experience hallucinations at least once a week (see Figure 2 for flow diagram of protocol).

### Questionnaires

The baseline questionnaire administered as part of a broader study on the psychosocial impact of CBS included demographic and clinical characteristic information and characteristics of and attitudes towards hallucinations, developed and validated by Cox and ffytche.^5^ This published data collected previously^12^ was used to characterise participants for this analysis. To ensure the data remained reflective of the participant's current experience of CBS, they were asked during recruitment whether their hallucination characteristics (e.g. frequency, length, perceived severity) had changed since completing the baseline questionnaire, and any updates were recorded.

The follow-up questionnaire was sent to all participants after having access to the podcast for ∼3-weeks. The participants were surveyed to assess if they had initiated any behavioural changes towards hallucinations. The primary outcome measure was captured by the question: ‘*Have you made any changes to how you respond to your hallucinations since listening to the podcast?’* (Yes/No). To explore shifts in attitude or behaviour, participants were asked: ‘*Were you inspired to try any of the interventions featured in the podcast?’* (Yes/No). To assess which strategies participants found helpful, participants were asked: ‘*Out of the following interventions, which have you tried since hearing the podcast?’* Responses to this question were rated on a 5-point Likert scale, ranging from ‘Did not try it’ to ‘Helped a lot’.

Other secondary outcome measures included questions designed to explore different aspects of the participants’ experiences. Participants were asked whether they had experienced any changes in the characteristics of their hallucinations, such as frequency or length. Additionally, participants were asked whether they found the podcast useful, enjoyable, and whether they had told anyone new about their symptoms since listening to the podcast.

While the survey used in this analysis was part of a broader study and featured additional questions, the focus of this study was on these outcome measures. The full baseline questionnaire and the follow-up questionnaire are available in Supplemental Materials. Participant information was anonymised before being entered into a secure computer database.

### Power calculation

As this is the first study to examine the effectiveness of two educational podcasts on participants’ behavioural responses with CBS, there are no existing data to inform anticipated change or effect size. Consequently, a pragmatic sample size aim of 50 participants per arm (100 participants in total) was determined. This sample size provides 80% power to detect a standardised mean difference of 0.6 between any pair of groups at a two-sided significance level of 0.05. This difference represents a medium-to-large effect.

### Statistical analysis

All data analyses were done in R version 4.2.2 (http://www.r-project.org/) under R Studio (RStudio, Boston, MA, USA) including use of the ggplot2 and likert package. The primary outcome was the proportion of participants reporting a change in how they respond to hallucinations (Yes/No) following the podcast. Group differences between the interaction and education podcast arms were assessed using Pearson's chi-squared test. Secondary analyses examined group differences in other binary outcomes (e.g. feeling inspired to try an intervention, disclosing symptoms to someone new), also using Pearson's chi-squared tests. Likert-scale responses regarding the use and perceived helpfulness of individual interventions were summarised descriptively and visualised using the likert package. 95% Confidence Intervals (CIs) are reported for proportion data.

## Results

### Demographics and clinical characteristics

Fifty-four people with CBS (74% female; 80%; aged >65 years) were deemed eligible for inclusion in the study, received the podcast and completed the final questionnaire (see Table 1). For details of the exclusions and participant attrition with associated reasons, see Figure 2. The most common vision impairment in the cohort was AMD (n = 30; 55%) and 78% had binocular vision loss. Twenty-six participants received the intervention podcast and 28 received the control podcast.

**Table 1.** Demographics and clinical characteristics of population.

	Educational podcast (n = 28)	Interaction podcast (n = 26)	Whole cohort (n = 54)
18–35 years	0	1	1
36–50 years	2	2	4
51–65 years	4	2	6
Over 65 years	22	21	43
Female	20	20	40
Male	8	6	14
Age-related macular degeneration	19	15	34
Diabetic retinopathy	1	0	1
Glaucoma	4	4	8
Retinitis Pigmentosa	3	0	3
Cataracts	3	0	3
Tumour (brain or ocular)	1	2	3
Other^a^	4	4	8
Monocular impairment	9	3	12
Binocular impairment	19	23	42

a Other diagnosis include stroke (n = 1), Leber's hereditary optic neuropathy (n = 1) and unreported (n = 2) for education podcast recipients; stroke (n = 1), hydrocephalus (n = 1), Pseudo Xanthoma Elasticum (n = 1), retinal vein occlusion (n = 1), hemianopia (n = 1) for interaction podcast recipients.

The majority of participants (41% (95% CI: 28%, 55%)) reported hallucinations to last minutes (n = 13 (47%; 95% CI: 28%, 66%) education podcast; n = 9 interaction podcast (35%; 95% CI: 17%, 56%)) and 20 participants (37%; 95% CI: 24%, 51%) experienced them almost every day (n = 9 education podcast (32%; 95% CI: 16%, 52%); n = 11 interaction podcast (42%; 95% CI: 23%, 63%)). Most participants experienced complex hallucinations (n = 22 education podcast (79%; 95% CI: 59%, 92%), n = 21 interaction podcast (81%; 95% CI: 61%, 93%)) while only 11 participants (20%; 95% CI: 10%, 34%) experienced simple hallucinations only.

### Participant recruitment and attrition

Participants were recruited between March 2022 and March 2023. In this study 110 people were recruited and randomised. Approximately 50% of participants were lost to follow-up, with the largest attrition (62%) occurring between receiving the podcast and receiving the questionnaire. The majority of participants who were lost to follow-up provided only an email address for contact and received study materials and the podcast link via email. In contrast, those who provided a phone number or postal address experienced lower rates of attrition, as they were contacted directly by a member of the research team (BH).

### Primary results

#### Behaviour change post-podcast

The majority of participants (n = 43; 80% (95% CI: 66%, 89%)) reported they did not change how they responded to hallucinations after receiving the podcast. This included 77% (n = 20) of those in the interaction podcast group (95% CI: 56%, 91%) and 82% (n = 23) in the education podcast group (95% CI: 63%, 94%). There was no statistically significant difference in behaviour change between the two groups (*p* = 0.80), representing the primary outcome of the study. The difference in the proportions of participants reporting no change in response between the podcast groups was −5% (95% CI: −34% to 24%), indicating that the difference between the two groups is not statistically significant.

### Secondary results

#### Inspired behaviour change

Half the participants said they were *inspired* to try the interventions suggested in the podcast. This included 46% (n = 12) of participants in the interaction podcast (95% CI: 27%, 67%) and 57% (n = 16) in the education podcast (95% CI: 37%, 76%). There was no difference in willingness to try the management techniques between the groups (*p* = 0.70). The difference in the proportions of participants reporting willingness to try the strategies between the two groups was −11% (95% CI: −39% to 7%), indicating that the difference between the two groups is not statistically significant.

#### Management strategies tried

**Figure 3.**
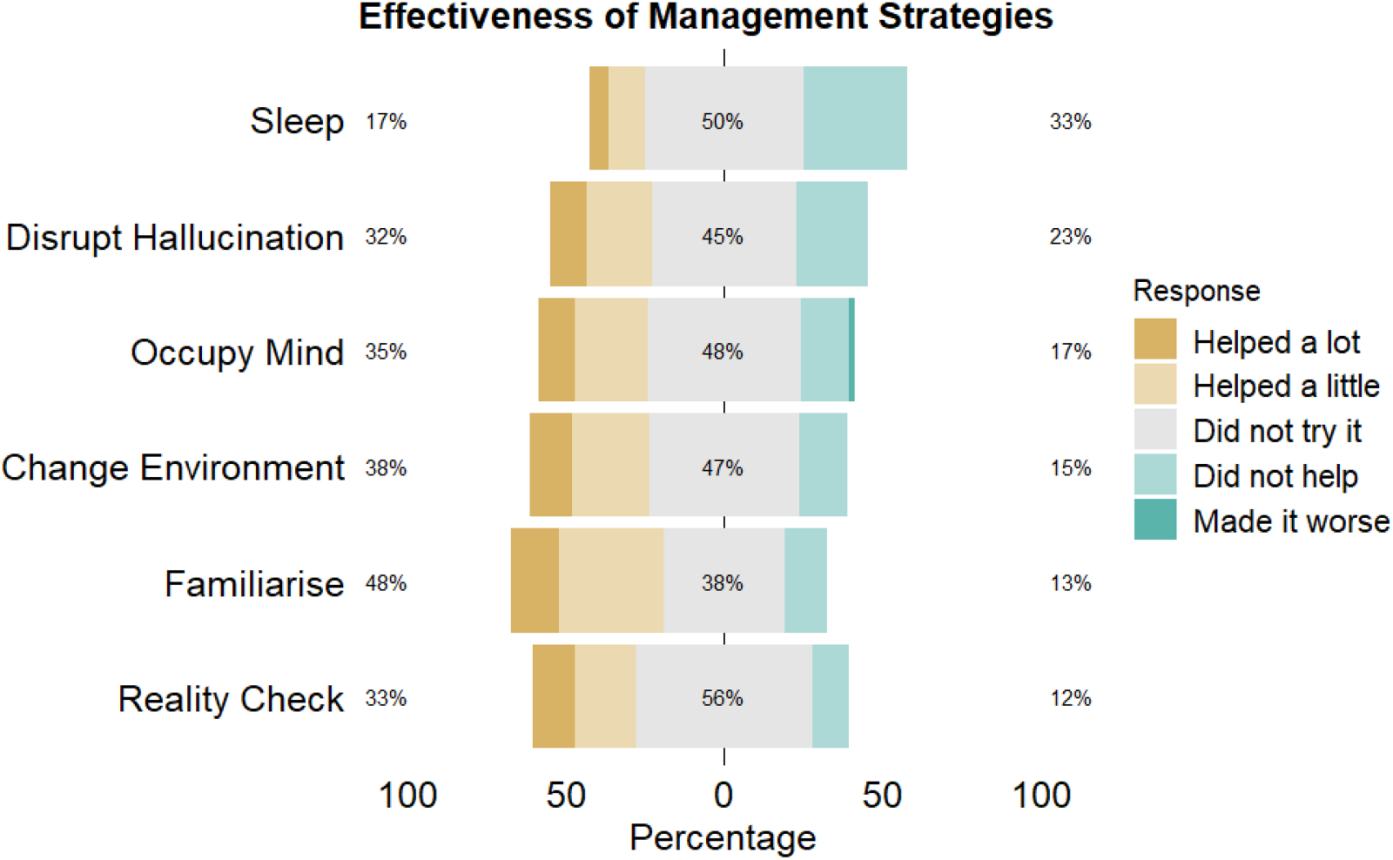
The plot displays participant ratings of the management strategies for their effectiveness. Responses were categorised as ‘helped a lot’, ‘helped a little’, ‘did not try it’, ‘did not help’ and ‘made it worse’.

While 80% (95% CI: 66%, 89%) of participants reported no change in how they responded to their hallucinations after listening to the podcast, half of participants indicated they had used the self-management strategies suggested. Among those who indicated they tried the strategies, 48% (95% CI: 34%, 62%) found that familiarising themselves with their hallucinations was the most helpful, making it the most attempted strategy. This was followed by changing their environments (38% (95% CI: 25%, 52%)), keeping their mind occupied (35% (95% CI: 22%, 49%)), reality checks (33% (95% CI: 20%, 47%)), and disrupting the hallucination (32% (95% CI: 20%, 46%)). Attempting to improve sleep was the least effective strategy, with 33% (95% CI: 20%, 47%) of participants reporting no impact, and only 17% (95% CI: 8%, 30%) finding it beneficial. One participant reported that trying to occupy their mind actually worsened their symptoms (see Figure 3 for more details).

#### Changes in frequency and length

**Figure 4.**
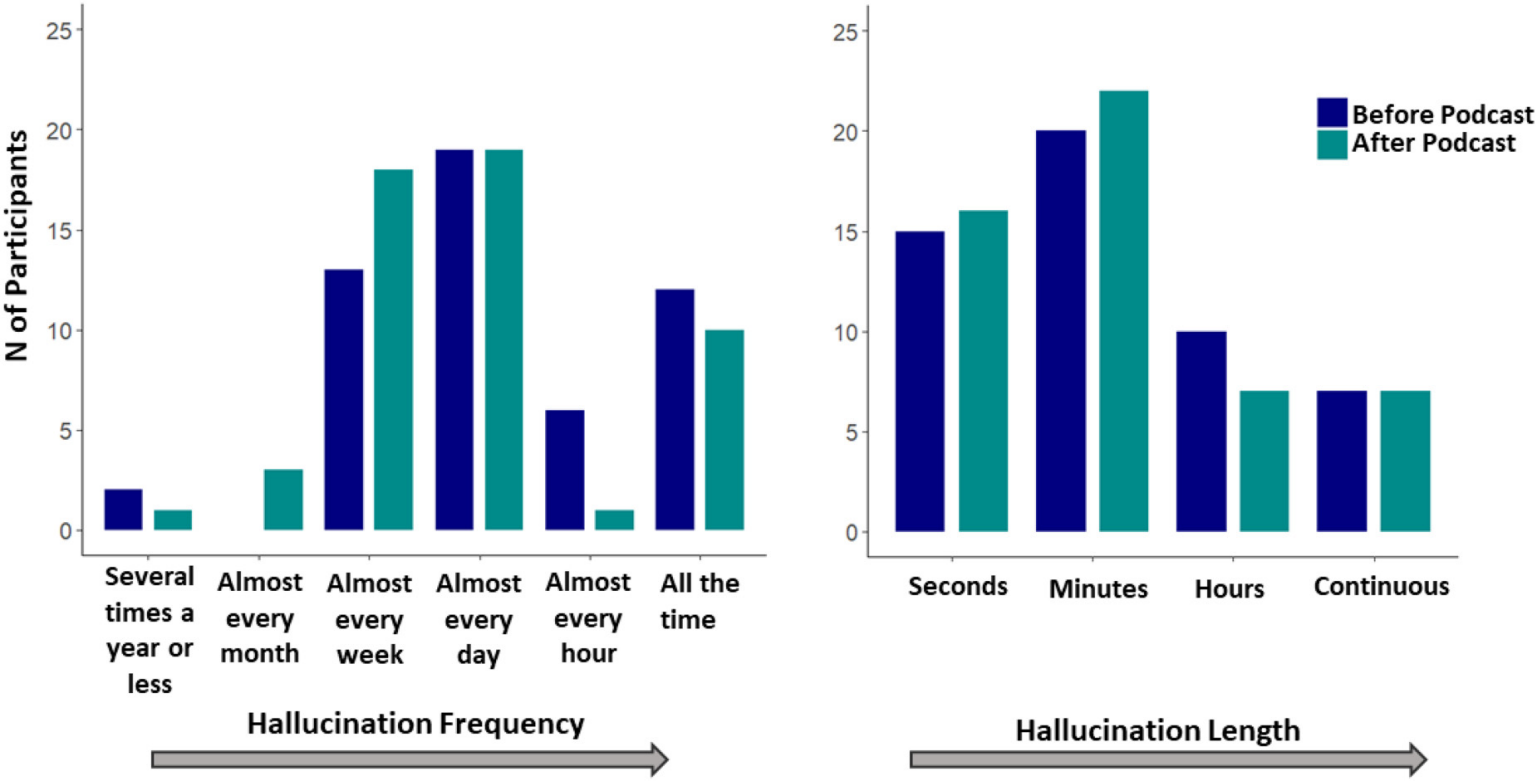
Plots depicting reported hallucination frequency (a) and length (b) before and after podcast.

*Note:* There were no statistically significant changes pre- to post-intervention in hallucination frequency or duration.

Twenty-nine participants (54% (95% CI: 40%, 67%)) reported no change in hallucination frequency since listening to the podcast. However, 19 people (35% (95% CI: 23%, 49%)) reported less frequent hallucinations after listening to the podcast and four participants (7% (95% CI: 2%, 17%)) reported an increase in hallucination frequency. Thirty-three participants (61% (95% CI: 47%, 74%)) reported no change in hallucination length, while 13 participants (24% (95% CI: 13%, 38%)) reported that hallucinations had reduced in length and eight participants (15% (95% CI: 6%, 27%)) report an increase in hallucination length. Overall, there were no statistically significant differences in change in hallucination frequency (*p* = 0.90) or length (*p* = 0.30) between the interaction podcast recipients and the educational podcast recipients (see Figure 4).

#### Feedback on podcast

Overall, 33 participants (61% (95% CI: 47%, 74%)) found the podcast useful, and 23 participants (42% (95% CI: 29%, 57%)) found it enjoyable. While there were no statistically significant differences between the groups’ perceived usefulness (*p* = 0.28) or self-reported enjoyment (*p* = 0.05) of the podcasts, the latter approached significance but did not meet the predefined alpha level of <0.05. Only 27% (95% CI: 12%, 48%) of people who received the interaction strategy rated it as enjoyable while the majority of the group rated it as ‘neutral’.

#### Sharing symptoms with new people

Since listening to the podcast, 34 participants (63% (95% CI: 49%, 76%)) reportedly spoke to new people about their symptoms and experience managing CBS, with no significant difference between the two podcast groups (*p* = 0.10).

## Discussion

This study investigated the effectiveness of two podcast-based educational approaches to support management of CBS hallucinations. The primary objective was to determine whether an interaction podcast, which promoted active engagement with hallucinations, resulted in different behavioural responses compared to an educational podcast that offered information on CBS and management strategies.

Our findings indicated that while the podcasts were felt ‘useful’ by more than half of the participants, 80% reported no change in their behaviour. Neither podcast lead to significant behaviour change in adopting new management strategies for CBS, nor resulted in any changes in hallucination length or duration. However, approximately half of participants intended to use suggested strategies, and 63% disclosed their symptoms to new people after listening.

### Interpretation and theoretical context

The lack of reported behaviour change in 80% of participants highlights a common issue in behaviour change interventions: the gap between intention and action.^13^ While the podcast increased awareness and motivation, it lacked the necessary components to translate this into practical application. According to the Theory of Reasoned Action (TRA), behavioural intention is influenced by an individual's attitude towards the behaviour and subjective norms.^14^ While the podcast may have positively influenced participants’ attitudes to adopt new management strategies, it did not ensure that these intentions led to actual behaviour change. The Theory of Planned Behaviour extends TRA by incorporating perceived behavioural control as a determinant of both intention and behaviour.^15^ This additional component suggests that even if individuals have favourable attitudes and perceive supportive social norms, they also need to believe they have control over performing the behaviour. In this study, participants might have lacked confidence in their ability to manage their hallucinations effectively or faced practical barriers, which impeded the translation of intention into action.

Furthermore, the lack of significant differences between the interaction and education podcasts suggests that presenting a management strategy in a podcast format might not be sufficient to induce behaviour change. Future interventions could aim to identify and mitigate these barriers through offering participants continuous support and regular appointments with healthcare professionals, evidenced to aid in successful behaviour change.^16^ Other mechanisms like workshops, personalised follow-ups or coaching could also be explored.

These findings are consistent with Robins et al., who indicated that while there is evidence showing that podcasts may enhance health knowledge and facilitate changes in behaviours like increased physical activity, the impact on psychological outcomes such as depression and anxiety has been less consistent.^11^ Similarly, while participants reported increased awareness and engagement, outcomes related to CBS management did not show significant improvement, indicating that podcasts may be more effective in raising awareness rather than inducing substantial behaviour change.

The findings can also be analysed through the lens of the Health Belief Model (HBM). According to the HBM, individuals with CBS are more likely to engage in a health behaviour (e.g. management strategies) if they perceive the condition as serious, believe in the effectiveness of a strategy and perceive minimal barriers to action.^17^ In this study, the podcast appears to have fallen short in addressing the perceived barriers to adopting new management strategies and in enhancing participants’ confidence in their ability to effectively manage their hallucinations. Thus, while the educational content was effective in raising awareness and concern about CBS, it did not empower individuals to take action to adopt the suggested strategies. This is potentially due to the fact that participants don’t *know* that these behaviour changes will make a difference, as we lack empirical evidence for their use. Future studies should focus on boosting self-efficacy, potentially through more interactive elements to translate intention into actual behaviour change.

### Existing knowledge and participant background

Notably, many participants reported using strategies after listening to the podcasts, despite not being asked beforehand if they already employed them. This omission was intentional, to avoid influencing behaviour during the study. However, given that 80% of participants reported no changes in their behaviour, yet nearly half indicated they had tried techniques such as familiarising themselves with their hallucinations, it is likely that many of these strategies were already part of their management repertoire before the study.

This is further supported by the fact that participants were primarily recruited from charity groups, which may have already provided them with greater awareness of available strategies. If the podcast had been tested on a group less familiar with self-management techniques – such as individuals who were newly diagnosed or less involved in support networks – the uptake of the strategies due to the podcasts may have been higher.

### Timing and measurement of change

Willingness to engage with the hallucination management strategies but not recording having done so, could indicate that perhaps the 3-week window in which participants first received the podcast and filled in the questionnaire may not have been a long enough window for a quantifiable behaviour change to occur. Further surveying of the participants at a later stage would be a potential option to explore this further, and is a potential future avenue for this research.

### Symptom disclosure

In addition, the podcast served as an effective educational tool, increasing awareness and encouraging symptom disclosure.^11^ About 63% of participants reported sharing their CBS experience with new people after listening to the podcast, suggesting they felt empowered to discuss their symptoms, much like the patient featured in the podcast who shared their own experience. While this was not a controlled comparison, this suggests a reduction in stigma and an improvement in social support networks the change was reported within the study period and may reflect a genuine pre–post outcome.

### Participant-led insights on strategy use

To the author's knowledge, this is the first study to investigate the effectiveness of management strategies for hallucinations in CBS. Various management strategies have been proposed to mitigate hallucinations in CBS patients, including techniques such as actively interacting with the hallucinations through touching or verbal communication. However, given that existing recommendations are based largely on anecdotal evidence,^8^ it remains unclear which interventions are most effective in managing CBS hallucinations.

This study recorded valuable information about self-management strategies currently utilised by people with CBS, highlighting those considered most effective, such as familiarising themselves with their hallucinations or changing their environment. Interestingly, the most reported strategy, familiarising with the hallucinations, was also the one most frequently cited as helpful. The process of familiarising oneself with hallucinations may help individuals gain a sense of control over their symptoms and reduce their emotional impact. This aligns with studies using psychological frameworks like Cognitive Behavioural Therapy used to manage psychosis, where individuals are encouraged to confront and make sense of their hallucinations.^18^ This approach has been shown to significantly improve psychological well-being by helping people reframe their hallucinations and develop coping strategies for better symptom management.^19^ Future research should systematically evaluate this and the other self-management techniques, considering their ease of implementation and overall effectiveness, to better inform patients and healthcare providers on the most reliable and effective strategies for managing CBS hallucinations.

### Study strengths

This study's strengths include the involvement of PPI during the study and podcast design ensured that the outcomes are relevant and meaningful to those directly affected by the research. Furthermore, to the authors’ knowledge, this study is the first to report on the management strategies used by a large cohort of people with CBS and to evaluate their perceived effectiveness. Another notable strength of this study is that the education-based podcast, which encouraged open discussions about hallucinations, will be made freely available. This will ensure broader accessibility, particularly for the charities that assisted in recruitment, as well as for any individuals who request it.

### Study limitations

A primary limitation was the sample size. Based on our power calculation, we aimed to recruit n = 50 participants per arm, enough to detect a medium-to-large effect with 80% power at a 5% significance level. Ultimately, we recruited n = 26 and n = 28 participants in the interaction and education groups, respectively. As a result, the study may have been underpowered. However, recruitment for CBS research is uniquely challenging due to the underreporting of the condition and stigma. These factors likely contributed to lower-than-anticipated enrolment and highlight the broader difficulties of conducting research in under-represented populations. Despite these challenges, we believe the data remain valuable in guiding future intervention design and feasibility efforts.

Many participants were lost to follow-up and this was a further limitation, particularly those recruited via email. In hindsight, requiring a phone number may have reduced attrition by facilitating more personal follow-ups. Another key limitation is the participant pool itself. Most were already involved in CBS support networks, likely familiar with existing strategies, and potentially less receptive to adopting new ones. Furthermore, the short evaluation window may not have captured longer-term changes in behaviour.

Another limitation relates to the intervention design. Although both podcast versions included references to various management strategies, the difference in the interaction podcast was a brief final segment whereby the clinician and narrator encouraged use of the interaction technique. This subtle distinction may not have been enough to influence behaviour meaningfully. Future iterations could benefit from more consistent emphasis on the intended strategy throughout the content to support greater uptake.

The cohort examined was predominantly female. In a recent systematic literature review of CBS literature in relation to AMD, it was concluded from 18 studies of >4000 participants that female gender was associated with CBS in people with AMD, therefore it is perhaps unsurprising our AMD-dominant cohort reflected this greater prevalence.^20^ This finding is further supported by a recent systematic review and meta-analysis of CBS prevalence across ophthalmic populations more broadly, which also found higher prevalence among females.^21^ Lastly, the absence of ethnicity data in this study limits the generalisability of the results. To address this limitation, future studies should prioritise working with ethnically diverse populations and collecting ethnicity data.

## Conclusion

In conclusion, while the podcast intervention increased awareness among individuals with CBS and the people around them, it did not lead to behaviour change in management strategies. Future research should focus on identifying and addressing barriers to behaviour change and exploring more interactive or supportive formats to enhance the effectiveness of educational podcasts.

## Supplemental Material

sj-docx-1-dhj-10.1177_20552076251382830 - Supplemental material for Podcast-driven insights into Charles Bonnet Syndrome: Impact on self-management strategies and communication Supplemental material, sj-docx-1-dhj-10.1177_20552076251382830 for Podcast-driven insights into Charles Bonnet Syndrome: Impact on self-management strategies and communication by Bethany E Higgins, Deanna J Taylor, Sonali Dave, Sarah Sowerby, David P Crabb and Tamsin Callaghan in DIGITAL HEALTH

sj-docx-2-dhj-10.1177_20552076251382830 - Supplemental material for Podcast-driven insights into Charles Bonnet Syndrome: Impact on self-management strategies and communication Supplemental material, sj-docx-2-dhj-10.1177_20552076251382830 for Podcast-driven insights into Charles Bonnet Syndrome: Impact on self-management strategies and communication by Bethany E Higgins, Deanna J Taylor, Sonali Dave, Sarah Sowerby, David P Crabb and Tamsin Callaghan in DIGITAL HEALTH
