# Supplementary material for: Podcast-driven insights into Charles Bonnet Syndrome: Impact on self-management strategies and communication
Source: Digit Health. 2025 Nov 18;11:20552076251382830. doi: 10.1177/20552076251382830 (PMC12627372; doi:10.1177/20552076251382830)
Supplement: sj-docx-2-dhj-10.1177_20552076251382830 - Supplemental material for Podcast-driven insights into Charles Bonnet Syndrome: Impact on self-management strategies and communication [file sj-docx-2-dhj-10.1177_20552076251382830.docx]

**Questionnaire Introduction**

Thank you so much for agreeing to be part of this new research study entitled **‘An investigation into the prevalence and nature of hallucinations in Charles Bonnet Syndrome (CBS)’**

In order to evaluate our podcast, your answers must be as accurate as possible.

There are 3 sections in total. Please take as much time as you need to answer each question. All your answers are confidential. Please circle your answers.

Date questionnaire filled in: _________

**Please enter your date of birth:**______________________

**Part A**

**This set of questions is about your experience of CBS since listening to the podcast**

1. **When was your last hallucination? (years/months/days)**

(Please state in the box below)

**2. Do you consider your hallucinations to have stopped?**

Yes

No

**If yes, please skip to Part B, page 6.**

**3. How long does each hallucination last on average since listening to the podcast?**

Seconds

Minutes

Hours

Continuous

**4. When at their worst, how frequently do the hallucinations occur since listening to the podcast?**

**(Circle one that best applies)**

Something was occurring all the time

I had a hallucination almost every hour

I had a hallucination almost every day

I had a hallucination most weeks

I had a hallucination most months

I had a hallucination several times a year or less

**5. What is your reaction to Charles Bonnet Syndrome now since listening to the podcast? (Circle all that apply)**

Amused

Curious

Intrigued

Startled

Frightened

Terrified

Indifferent

Frustrated

Other (please state in the box below)

**6. Charles Bonnet Syndrome has the following effect on my life since listening to the podcast (Circle the one that best applies)**

A very negative effect

Fairly negative

No real effect

A fairly pleasant effect

A very pleasant effect

**7. Who have you told about your hallucinations since listening to the podcast? (Circle all that apply)**

Medical professionals

Spouse

Other family

Friends

Other people with macular disease

No one

Other (please state in the box below)

**This is the end of Part A, please begin Part B**

**Part B**

**This set of questions is about your thoughts on the podcast.**

1. **How enjoyable did you find the podcast?**

Very enjoyable

Fairly enjoyable

Neutral

Not very enjoyable

Not at all enjoyable

1. **How useful did you find the podcast?**

Very useful

Fairly useful

Neutral

Not very useful

Not at all useful

1. **Have you made any changes to how you respond to your hallucinations since listening to the podcast?**

No

Yes (Please state these changes in the box)

1. **Were you inspired to try any of the interventions featured in the podcast?**

Yes

No

1. **Out of the following interventions, which have you tried since hearing the podcast? (Circle all that apply and how you feel it helped)**

**Change things** (like changing the light level)

Helped a lot

Helped a little

Did not help

Made it worse

Did not try it

**Occupy your mind** (like putting the tv or radio on, or moving rooms)

Helped a lot

Helped a little

Did not help

Made it worse

Did not try it

**Sleep** (getting enough sleep)

Helped a lot

Helped a little

Did not help

Made it worse

Did not try it

**Getting to know them** (familiarising yourself with the hallucinations)

Helped a lot

Helped a little

Did not help

Made it worse

Did not try it

**Reality check** (asking yourself, is this too detailed to be real?)

Helped a lot

Helped a little

Did not help

Made it worse

Did not try it

**Disrupting the hallucination** (reaching out and moving it or waving it away)

Helped a lot

Helped a little

Did not help

Made it worse

Did not try it

1. **Were there any other parts of the podcast you found helpful?**

No

Yes (Please state in the box)

1. **Has your attitude towards your CBS changed after listening to the podcast?**

No

Yes (Please state how in the box)

1. **How do you feel about a podcast being used to communicate educational material about CBS?**

Please state answer in the box

**This is the end of Part B, please begin Part C**

**Part C**

**This section consists of a number of words that describe different feelings and emotions. Read each item and then circle the appropriate answer that describes the way you feel generally, i.e. on average about life.**

*For example:*

**Interested**

Not at all/Very slightly A little Moderately Quite a bit Extremely

**Please indicate an answer for all the questions and do not leave any blank.**

1. **Interested**

Not at all/Very slightly A little Moderately Quite a bit Extremely

1. **Distressed**

Not at all/Very slightly A little Moderately Quite a bit Extremely

1. **Excited**

Not at all/Very slightly A little Moderately Quite a bit Extremely

1. **Upset**

Not at all/Very slightly A little Moderately Quite a bit Extremely

1. **Strong**

Not at all/Very slightly A little Moderately Quite a bit Extremely

1. **Guilty**

Not at all/Very slightly A little Moderately Quite a bit Extremely

1. **Scared**

Not at all/Very slightly A little Moderately Quite a bit Extremely

1. **Hostile**

Not at all/Very slightly A little Moderately Quite a bit Extremely

1. **Enthusiastic**

Not at all/Very slightly A little Moderately Quite a bit Extremely

1. **Proud**

Not at all/Very slightly A little Moderately Quite a bit Extremely

1. **Irritable**

Not at all/Very slightly A little Moderately Quite a bit Extremely

1. **Alert**

Not at all/Very slightly A little Moderately Quite a bit Extremely

1. **Ashamed**

Not at all/Very slightly A little Moderately Quite a bit Extremely

1. **Inspired**

Not at all/Very slightly A little Moderately Quite a bit Extremely

1. **Nervous**

Not at all/Very slightly A little Moderately Quite a bit Extremely

1. **Determined**

Not at all/Very slightly A little Moderately Quite a bit Extremely

1. **Attentive**

Not at all/Very slightly A little Moderately Quite a bit Extremely

1. **Jittery**

Not at all/Very slightly A little Moderately Quite a bit Extremely

1. **Active**

Not at all/Very slightly A little Moderately Quite a bit Extremely

1. **Afraid**

Not at all/Very slightly A little Moderately Quite a bit Extremely

**You have finished the Questionnaire.**

**Please check you have filled in all questions.**

**Thank you for your time.**
